# Diagnostic evaluation and ablation treatments assessment in hepatocellular carcinoma

**DOI:** 10.1186/s13027-021-00393-0

**Published:** 2021-07-19

**Authors:** Vincenza Granata, Roberta Grassi, Roberta Fusco, Andrea Belli, Carmen Cutolo, Silvia Pradella, Giulia Grazzini, Michelearcangelo La Porta, Maria Chiara Brunese, Federica De Muzio, Alessandro Ottaiano, Antonio Avallone, Francesco Izzo, Antonella Petrillo

**Affiliations:** 1 Division of Radiology, Istituto Nazionale Tumori IRCCS Fondazione Pascale – IRCCS di Napoli, Naples, Italy; 2 Division of Radiology, Università degli Studi della Campania Luigi Vanvitelli, Naples, Italy; 3 Italian Society of Medical and Interventional Radiology SIRM, SIRM Foundation, Milan, Italy; 4 Medical Oncology Division, Igea SpA, Naples, Italy; 5 Division of Hepatobiliary Surgical Oncology, Istituto Nazionale Tumori IRCCS Fondazione Pascale – IRCCS di Napoli, Naples, Italy; 6 Department of Medicine, Surgery and Dentistry, University of Salerno, Salerno, Italy; 7 Radiology Division, Azienda Ospedaliero-Universitaria Careggi, Florence, Italy; 8 Department of Radiology, UOC San Severo Hospital, San Severo, Italy; 9 Department of Medicine and Health Sciences “Vincenzo Tiberio”, University of Molise, Campobasso, Italy; 10 Abdominal Oncology Division, Istituto Nazionale Tumori IRCCS Fondazione Pascale – IRCCS di Napoli, Naples, Italy

**Keywords:** HCC, Ultrasound, Computed tomography;magnetic resonance imaging, Radiomics, Ablation treatment assessment

## Abstract

This article provides an overview of diagnostic evaluation and ablation treatment assessment in Hepatocellular Carcinoma (HCC). Only studies, in the English language from January 2010 to January 202, evaluating the diagnostic tools and assessment of ablative therapies in HCC patients were included. We found 173 clinical studies that satisfied the inclusion criteria.

HCC may be noninvasively diagnosed by imaging findings. Multiphase contrast-enhanced imaging is necessary to assess HCC. Intravenous extracellular contrast agents are used for CT, while the agents used for MRI may be extracellular or hepatobiliary. Both gadoxetate disodium and gadobenate dimeglumine may be used in hepatobiliary phase imaging. For treatment-naive patients undergoing CT, unenhanced imaging is optional; however, it is required in the post treatment setting for CT and all MRI studies. Late arterial phase is strongly preferred over early arterial phase. The choice of modality (CT, US/CEUS or MRI) and MRI contrast agent (extracelllar or hepatobiliary) depends on patient, institutional, and regional factors. MRI allows to link morfological and functional data in the HCC evaluation. Also, Radiomics is an emerging field in the assessment of HCC patients.

Postablation imaging is necessary to assess the treatment results, to monitor evolution of the ablated tissue over time, and to evaluate for complications. Post- thermal treatments, imaging should be performed at regularly scheduled intervals to assess treatment response and to evaluate for new lesions and potential complications.

## Introduction

Primary liver cancer is the sixth most commonly diagnosed cancer and the third leading cause of cancer death worldwide in 2020, with approximately 906,000 new cases and 830,000 deaths. Rates of both incidence and mortality are 2 to 3 times higher among men than among women in most regions, and liver cancer ranks fifth in terms of global incidence and second in terms of mortality for men. Incidence rates among men are 2.4-fold greater in transitioned countries, but the highest rates are observed mainly in transitioning countries, with the disease being the most common cancer in 11 geographically *differents* countries in Eastern Asia, South-Eastern Asia, and Northern and Western Africa [1, 2].

Primary liver cancer includes hepatocellular carcinoma (HCC) (comprising 75–85% of cases) and intrahepatic cholangiocarcinoma (10–15%), as well as other rare types. The main risk factors for HCC are chronic infection with hepatitis B virus (HBV) or hepatitis C virus (HCV), aflatoxin-contaminated foods, heavy alcohol intake, excess body weight, type 2 diabetes, and smoking [1]. The major risk factors appear to be in transition, with the prevalence of HBV and HCV declining and excess body weight and diabetes increasing in many regions [1]. Although the relevance of nonviral risk factors is becoming more important, the elimination of viral hepatitis remains the key strategy for primary prevention of liver cancer globally, seeing as HBV infection and HCV infection account for 56 and 20% of liver cancer deaths worldwide, respectively [1, 2].

Patients who are diagnosed at an early stage without metastasis are eligible for curative treatments, and hence, have a good prognosis in the range of 50–70% survival rate at 5-year [3–8]. However, the prognosis is poor when HCC is diagnosed at an advanced stage. Therefore, an early detection of HCC and an accurate characterization of focal liver nodule on patient at risk for HCC is mandatory for a suitable patient management [5].

Nowadays, the gold standard for HCC diagnosis is the needle biopsy, but this is an invasive and dangerous technique. The non-invasive criteria for HCC diagnosis are based on the presence of the specific vascular profile on imaging assessment, characterized by contrast uptake during arterial phase, defined as arterial hyperenhancement, and followed by washout in the venous/portal phase [9–12]. However, arterial hyperenhancement and wash out appearance have a sensitivity rate of 50–60% in lesion smaller than 2 cm [9]. Therefore, other functional parameters have been introduced in the detection and characterization of HCC nodules [9–13]. Diffusion Weighted Imaging (DWI) has been applied to liver imaging as an excellent tool for detection and characterization of focal liver lesions, increasing clinical confidence and decreasing false positives [13].

Tumor ablation is a minimally invasive approach that is commonly employed in the treatment of hepatic tumors [14]. Ablation therapy is considered a potential first-line treatment in many patients with small hepatocellular carcinomas (< 3 cm) [15] or an alternative for people who are not fit for surgical resection [16, 17]. Moreover, tumor ablation can also be useful as an adjuvant therapy or may provide an alternative strategy to surgery or be used in association with resection in case of patients unfit for surgical treatments [17–21]. Ablation therapies, as stand alone or in combination, have created a new challenge for radiologists, who should assess the response. The goal of locoregional therapy is inducing necrosis. Therefore, tumor shrinkage may not be apparent or may be absent with thermal approaches. Tumor physiologic features such as angiogenesis and hypoxia are more relevant to demonstrating tumor response in this setting, and thus require the development of new functional imaging biomarkers.

Our aim is to report an overview and update on diagnostic assessment in HCC patients.

## Methods

This narrative review is the result of autonomous study without protocol and registration number.

### Search criterion

Several electronic datasets were searched: PubMed (US National Library of Medicine, http://www.ncbi.nlm.nih.gov/pubmed), Scopus (Elsevier, http://www.scopus.com/), Web of Science (Thomson Reuters, http://apps.webofknowledge.com/) and Google Scholar (https://scholar.google.it/). The following search criteria have been used: “HCC” AND “Ultrasound”, “HCC” AND “Computed Tomography”, “HCC” AND “Magnetic Resonance Imaging”, “HCC” AND “Radiomics”, “HCC” AND “Li-RADS”, “HCC” AND “Ablative Therapies” AND “Assessment”.

The search covered the years from January 2010 to April 2021. Moreover, the reference lists of the found papers were analysed for papers not indexed in the electronic databases. All titles and abstracts were analysed. The inclusion criteria were clinical study (eg. retrospective analysis, case series, prospective cohort study) evaluating the diagnostic tools and safety and efficacy of ablative therapies in HCC patients. Articles published in the English language from January 2010 to January 2021 were included. Exclusion criteria were different topics, unavailability of full text, not sufficient data and case report, review, or letter to editors.

## Results

**Fig. 1:**
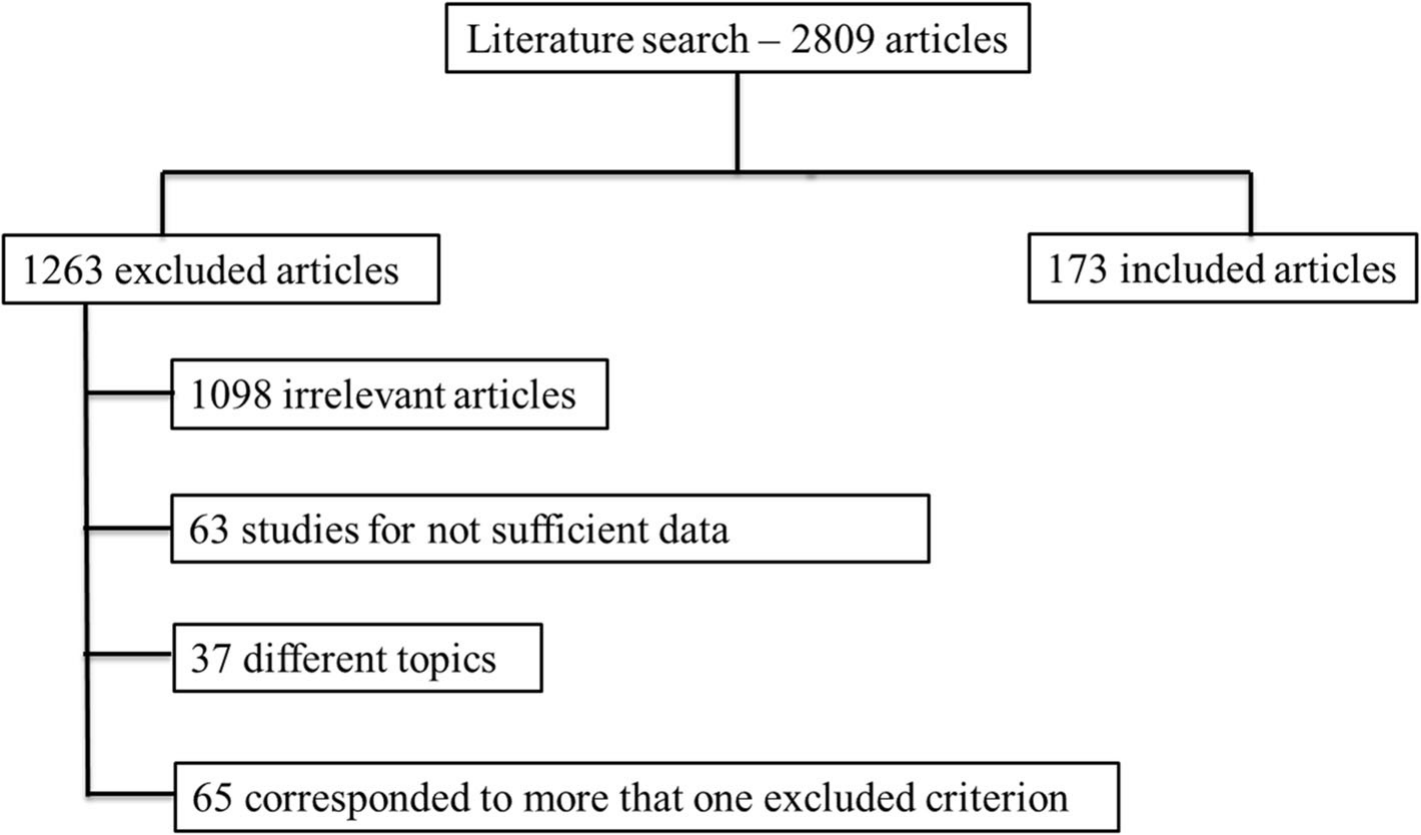
Included and excluded studies in systematic review

We identified 4220 potentially relevant references through electronic searches. After removing 2809 duplicates, we obtained 1411 references. We identified 25 references through scanning reference lists of the identified randomized trials that we added to the 1411 references previously selected (total number of scrutinized articles was 1436). We then excluded 1098 clearly irrelevant articles through screening titles and reading abstracts. We excluded 165 references for the reasons listed in the exclusion criteria. A total of 173 clinical trials met the inclusion criteria. The reference flow is summarized in the study flow diagram (Fig. 1).

## Discussion

### Diagnostic tools

#### Ultrasound

Ultrasound (US) is an imaging tool is cheap, non-invasive, non-irradiating and, thus, repeatable, suitable for patient disease monitoring [22–25]. Contrast-Enhanced Ultrasound (CEUS) imaging is an improved ultrasound-based technology, assuming the injection into the blood of a specific contrast agent, consisting of gas filled microbubbles [22]. The contrast agent spreads through the human body, emphasizing the vessel structure in the region of interest [22]. This technology leads to the highlighting of both large vessel flows, as well as of the microcirculation, being firstly implemented for hepatic tumor pathology, for abdominal emergencies and in order to recognize various tumor types [26–36]. The microbubbles of the contrast agent produce harmonic echoes, which are detected by the transducer. This behavior is significantly different from that of the usual US waves reflected by the tissues [22].

**Fig. 2:**
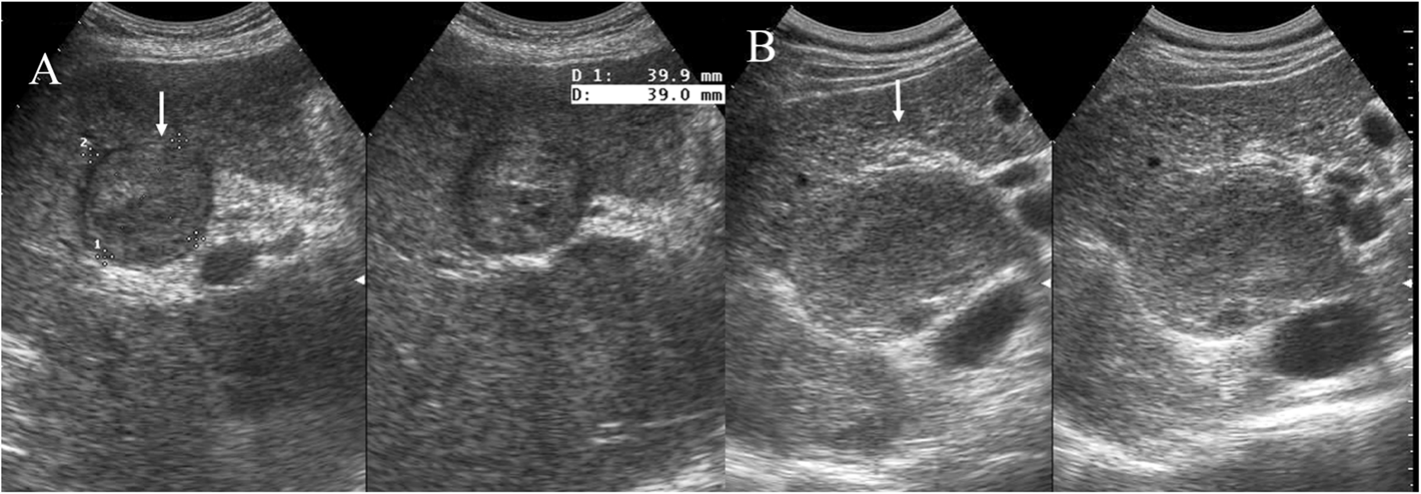
US assessment of HCC on IV a hepatic segment. The lesion (arrow) is hyper-isoechoic compared to liver parenchyma

**Fig. 3:**
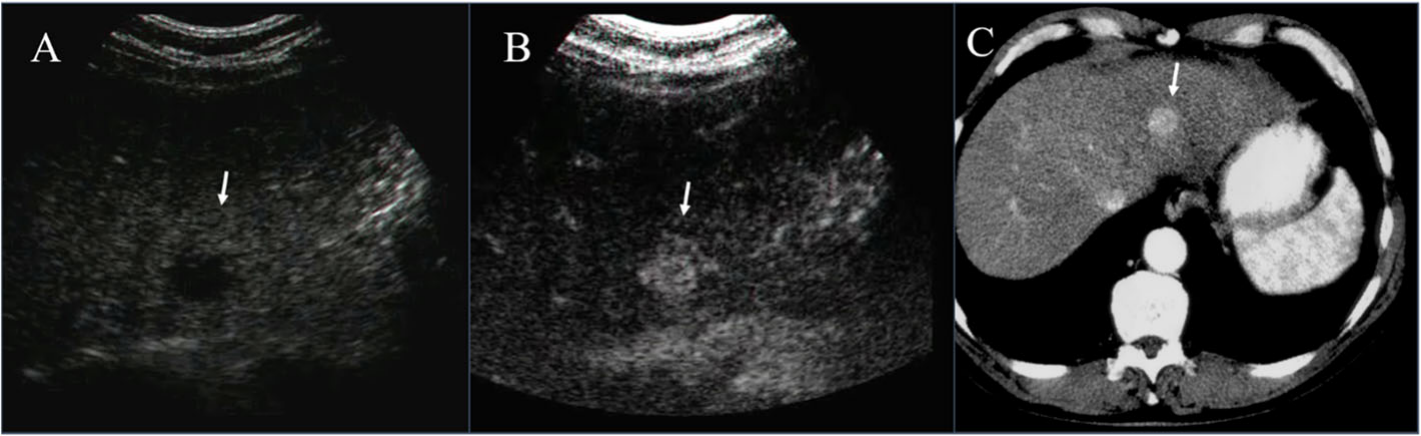
US (**A**), CEUS (**B**) and CT (**C**) assessment of HCC on II hepatic segment. The lesion in **B** and **C** (arrow) shows hyperenhancement during arterial phase of contrast study

**Fig. 4:**
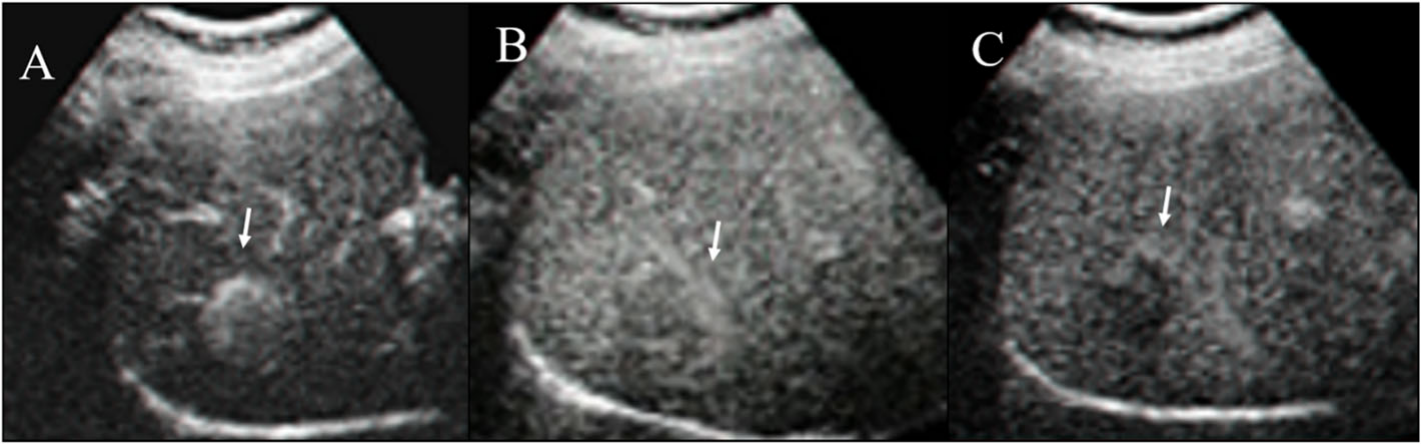
Ceus evaluation of HCC on VIII liver segment. During contrast study, the lesion (arrow) shows hyperenhancement during artierial phase (**A**), wash out during portal (**B**) and equilibrium (**C**) phase

Within B-mode US images, HCC appears, in more advanced evolution phases, as a well-defined region, of 3–5 cm in size, being hyperechogenic and often heterogeneous, due to the interleaving of fatty cells, necrosis, fibrosis and active growth tissue (Fig. 2) [22]. In CEUS images, HCC appears more highlighted, due to the dense and complex vessel structure that is specific to the malignant tumors (Fig. 3) [22]. The HCC tumors are usually hyperenhanced during the arterial phase, showing washout during the portal venous and delayed phases (Fig. 4) [22].

However, in many cases, within both B-mode US images and CEUS images, HCC is hardly distinguishable from the cirrhotic parenchyma.

#### Computed tomography

Currently, contrast-enhanced computed tomography (CE-CT) is commonly used for the noninvasive detection and characterization of focal liver lesions (FLLs) due to its high scanning speed and high-density resolution [37–47]. The appearances, especially the dynamic enhancement patterns of FLLs on CT imaging, are essential for categorizing lesions. With the careful evaluation of CT images, for most liver lesions the diagnosis can be achieved with a relatively high accuracy [37]. However, in current clinical practice, the evaluation of CT images is mainly performed by radiologists. The results are influenced by the radiologist’s experience and are generally subjective [37].

Multiphase contrast-enhanced computed tomography (CT) is often the first diagnostic imaging technique to diagnose HCC, and besides, Magnetic Resonance Imaging (MRI) represents the standard imaging method. For daily clinical routine, CT is the best available technique, and in contrast to CEUS it is examiner-independent [48]. Thus, CT allows a fast, reproducible and non very expensive examination. Especially for patients with reduced general state of health and restricted compliance, CT offers an adequate examination in contrast to MRI or CEUS, which are more dependent on the compliance of the patient [48].

**Fig. 5:**
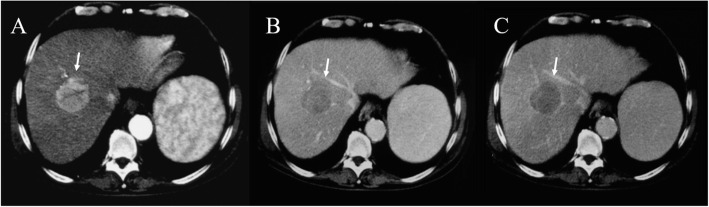
The same case of Fig. 4. CT assessment. During contrast study, the lesion (arrow) shows hyperenhancement during artierial phase (**A**), wash out during portal (**B**) and equilibrium (**C**) phase

**Fig. 6:**
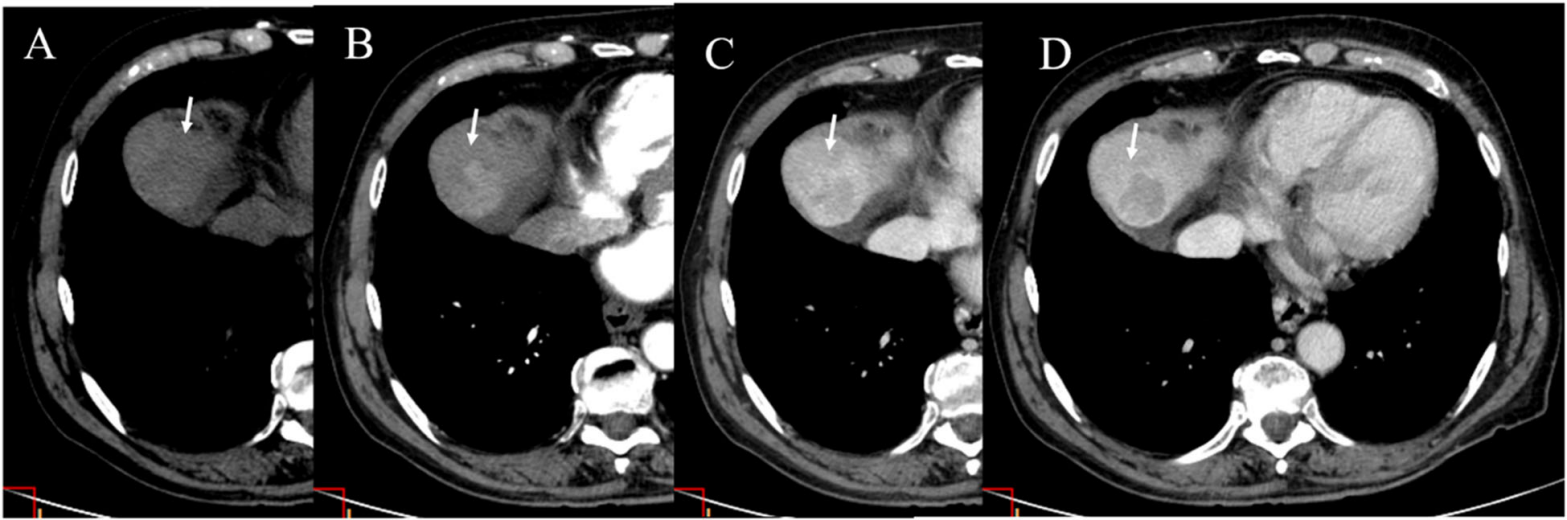
CT assessment of HCC on IVa hepatic segment. In (**A**) pre contrast phase. During contrast study, the lesion (arrow) shows hyperenhancement during artierial phase (**B**), wash out during portal (**C**) and capsule appearance in equilibrium phase (**D**)

**Fig. 7:**
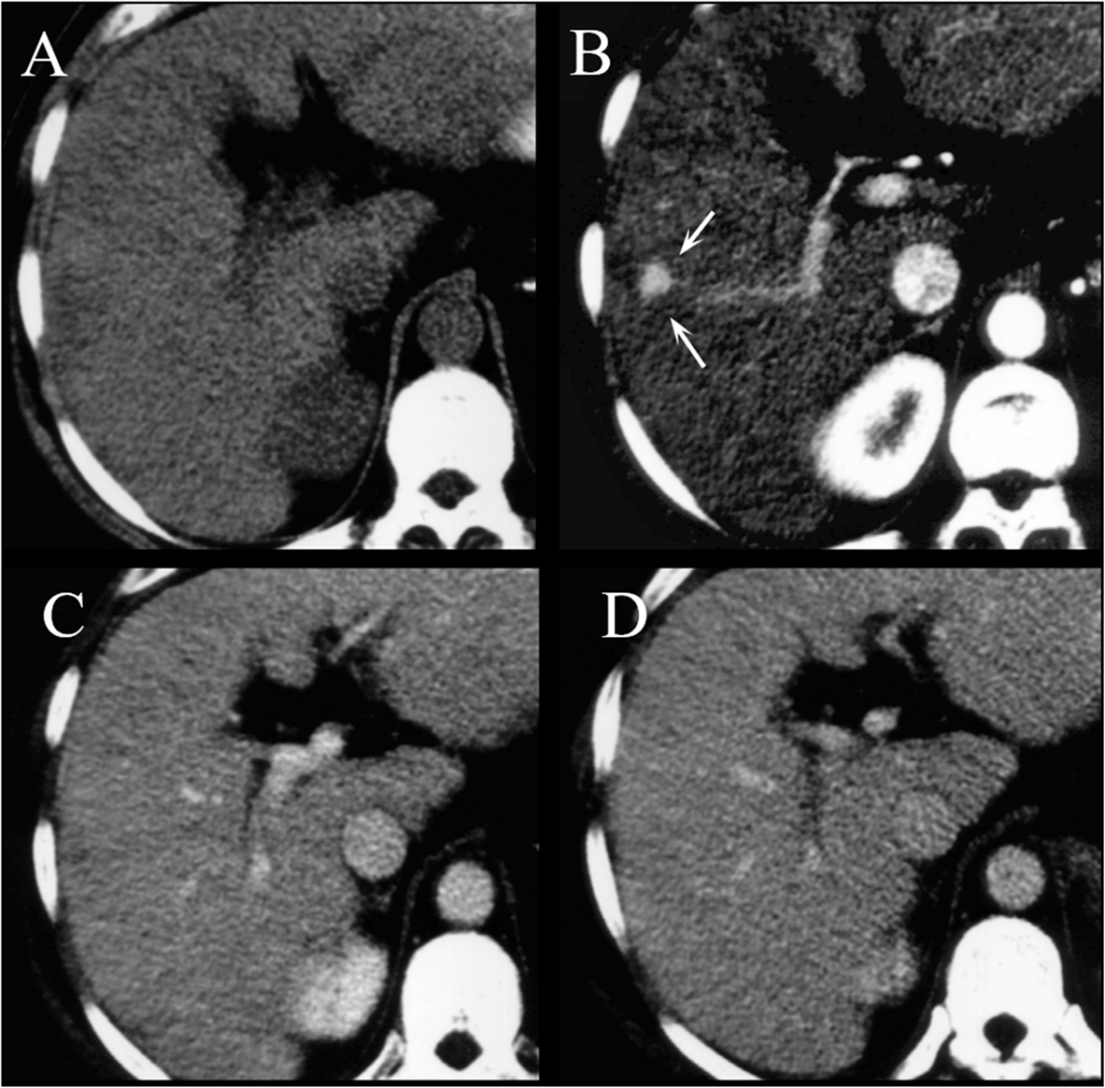
Early HCC on V hepatic segment: pre contrast phase (**A**), arterial phase (**B**), portal phase (**C**), equilibrium phase (**D**). The lesion (arrows) is deteced only during arterial phase of contrast study (**B**)

Classical CT protocol in the assessment of HCC involves multiphase study with non-contrast phase, arterial, portal/venous, and equilibrium phase [49]. The non-contrast phase is useful to detect hyperattenuation due to haemorrhage or hyperattenuating embolic agents like lipiodol before contrast administration, thus avoiding misinterpretation of arterial-phase hyperenhancement [49]. The arterial phase, which is characterized by full enhancement of the hepatic artery and beginning enhancement of the portal vein, is useful to detect hypervascular HCC [49]. The portal venous phase which is characterized by enhancement of hepatic veins as well as portal veins and the equilibrium phase imaging are useful for the differential diagnosis of HCC. Infact, the majority of HCCs shows washout of contrast medium in these phases (Figs. 5 and 6) [49]. However, early HCCs and dysplastic nodules are often iso- or hypo-vascularized with different enhancement patterns compared to hypervascularized advanced HCCs [49]. Early HCC can show iso-attenuation throughout all phases of dynamic CT (Fig. 7) [49].

Newer techniques have been introduced to increase lesion detection and characterization, such as dual-energy CT (DECT) with low-energy acquisition at 80 kVp instead of the frequently applied 120 kVp [45, 50–59]. DECT, which is based on simultaneously acquisition of two datasets at different energy levels, has the potential to generate virtual monochromatic images (VMIs). These images have higher contrast at lower energy levels because they use an approximation of energy levels closer to the k-edge of iodine at 33 keV [59]. Yoon et al. showed that low monoenergetic images (50 keV) allowed significantly better focal liver lesion conspicuity even after lowering both radiation and contrast media doses by 30%, compared with standard-dose in non obese patients [58]. Furthermore, 50 keV images obtained using the double low-dose CT protocol revealed higher image contrast, and better image quality than standard-dose iDose images at both arterial and portal venous phases [58]. Simultaneous radiation and contrast media dose reduction would particularly be useful for patients who undergo multiple CT scans. Patients with a suspicion of HCC or a history of HCC belong to this category owing to the demand for an intense follow-up strategy due to the high risk of HCC recurrence. However, radiation exposure is often underestimated in oncologic patients due to their relatively short life expectancy and the clinicians’ focus on the immense benefit of early detection of recurrence [58–61]. Indeed, patients with very early or early-stage HCCs who have a life expectancy of longer than 5 years, usually undergo multiple CT scans at 3- to 4-month intervals so as to detect recurrence [62]. Contrast media dose reduction should also be important for those patients because renal dysfunction is common in oncologic patients and cirrhotic patients [63]. Therefore, as suggested by Yoon, the reduction of radiation and contrast doses using DECT while maintaining image quality and diagnostic performance would hold great clinical value for patients with a high risk of HCCs who need to undergo multiple multiphasic liver CT scans [58].

Perfusion CT (pCT) is a non-invasive imaging tool based on contrast kinetics of tissue that supplies quantitative information on tissue hemodynamics. pCT measures dynamic changes in tissue iodine concentration over time, allowing for calculation of tissue-specific parameters, including blood flow (BF), blood volume (BV), time to peak concentration (TTP), vascular permeability surface area product (PS), and permeability (*K*trans), which can be used as surrogates for tumor vascularization, vascular immaturity, and perfusion pressure. Several researchers reported that pCT is argued to be a significant method for differentiating benign from malignant lesions, evaluating treatment response, and defining angiogenesis [64–66].

During the development of HCC from a low-grade dysplastic nodule to advanced HCC, the arterial blood supply and angiogenesis are increased. Quantifying HCC vascularity is important for evaluating tumor progression. pCT is highly promising as a functional vascular imaging technique. However, because of the increased radiation dose, CT perfusion of liver is largely unfulfilled clinically [64].

#### Magnetic resonance imaging

MRI is the imaging technique of choice in the assessment of focal liver lesions [67–73], particulary, in the diagnostic phase and during the follow up after treatment in HCC patients [74–82]. In the following section we will focus on several particular MRI features in the workup of HCC patients: morfological assessment, dynamic contrast enhancement (DCE)-MRI, diffusion-weighted imaging (DWI), Bold sequences.

### Morfological assessment

**Fig. 8:**
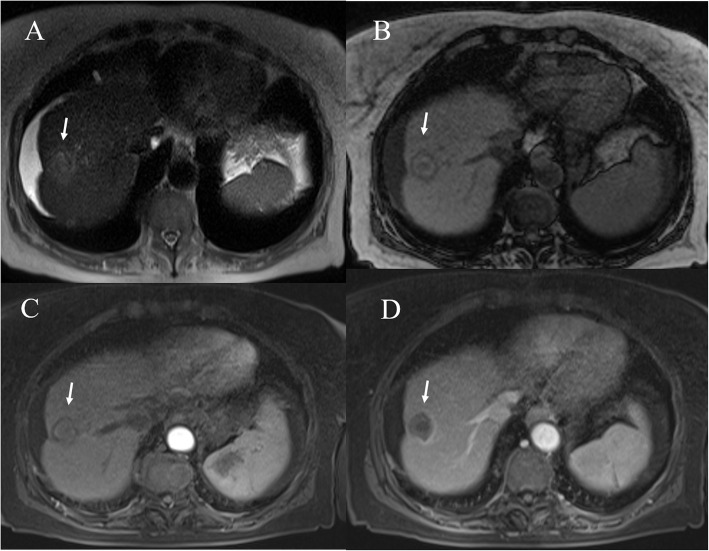
MRI assessment of HCC on II hepatic segment. The lesion show (arrow) hyperintense signal in T2-W sequence (**A**), hyperehancement during arterial phase (**B**), wash out appearance (**C**) during portal phase and nodule in nodule appearance in hepatobiliary phase (**D**)

MR imaging diagnosis of HCC is mainly based on assessment of vascularity, capsule appearance, and signal intensity (SI) during the hepatobiliary (EOB) phase. MR imaging also allows the assessment of morfological or ancillary features, that can be divided into those that favor the diagnosis of HCC specifically (intralesional fat, nodule-in-nodule architecture (Fig. 8), and mosaic architecture) and those that favor the diagnosis of malignancy but are not specific for HCC (mild-moderate T2 hyperintensity and lesional iron sparing) [9].

Intralesional fat is the presence of lipid within the nodule in higher concentration than in the hepatic parenchyma [9]. This feature can be detected at MR by observing signal loss in out-of-phase compared with in-phaseT1-W GRE images. In a patient at risk for HCC, the detection of intralesional fat in a solid nodule raises concern for malignancy or premalignancy. In fact, this feature does not establish the diagnosis of HCC, however, as the differential diagnosis includes high-grade dysplastic nodule and occasionally low-grade dysplastic nodule [9].

Mosaic architecture refers to the presence within a mass of randomly distributed internal nodules differing in enhancement, intensity, often separated by fibrous septa. This feature is characteristic of large HCCs and reflects the mosaic configuration observed at pathologic evaluation. It is unusual in tumors other than HCC [9].

T2-W hyperintensity is an ancillary imaging features. Park et al. showed that dysplastic nodules and HCCs cannot be distinguished on the basis of signal intensity characteristics on unenhanced MRI, since their signal intensities are similar on T1- and T2-W sequences [83]. However, dysplastic nodules are almost never hyperintense on T2-W, early HCCs are mostly isointense on T2-W, while higher grade (moderately or poorly) of HCC is associated with high SI on T2-W images, although the SI may also be related with tumor vascularity and peliotic changes [83]. Previous study demonstrated that T2-W hyperintensity was a highly specific marker of nodule malignancy, although poorly sensitive [84–87]. Golfieri et al. [87] showed that, compared to hypointensy on EOB phase, T2-W hyperintensity was a poor predictor of malignancy in the early stages of HCC. Conversely to Golfieri [87], Ouedraogo et al. [88] demonstrated that the addition of T2-W hyperintensity to the AASLD criteria increased the detection rate of HCC, especially for nodules smaller than 20 mm. In fact the sensitivity of MRI increased from 67.6 to 79%. Hwang et al. [89] compared the diagnostic performance of DWI and T2-W images in differentiating between hypovascular HCC and dysplastic nodules seen as hypointense nodules at hepatobiliary phase. They showed that hyperintensity on T2-W and DWI were significant features for differentiating hypovascular HCCs from dysplastic nodules. Kim et al. [90] evaluated the most predictive finding among hyperintensity on T2-W, DWI, washout, capsular enhancement, and hypointensity on gadoxetic acid-enhanced hepatobiliary phase images in the detailed characterization of arterial phase enhancing nodules 1 cm in diameter and smaller. They showed that for hypervascular lesions of 1 cm in diameter or smaller, T2-weighted images have the highest sensitivity among tests with an odds ratio statistically separable from 1 for differentiating HCC from benign hypervascular lesions 1 cm or smaller.

Lesional iron sparing refers to relative paucity of iron in a solid mass compared with that of background iron overloaded liver. This feature raises concern for premalignancy or malignancy because high-grade dysplastic nodules and HCCs characteristically are iron “resistant”. However it is not specific for high-grade dysplastic nodule or HCC, but other non-HCC malignancies may have this appearance [9].

### Dynamic contrast-enhanced (DCE)-MRI

DCE-MRI provides functional parameters on tumour perfusion, vessel permeability and ex- tracellular-extravascular space composition by assessing the changes in SI after the injection of a paramagnetic contrast medium*.* DCE- MRI may estimate data of the tumour vascular microenvironment, such as hypoxia and microvascular density, and also vascular changes induced by treatments. DCE-MRI can be evaluated by qualitatively, semi quantitatively and quantitatively method. Quantitative analysis involves the assessment of the pharmacokinetics of an administered contrast medium [91–95]. The most commonly used feature is the volume transfer constant, Ktrans, which represents the rate of contrast medium that moves from the blood to the extracellular space and relates to microvascular blood flow, vessel wall permeability, and vessel density. Ktrans has been shown to be correlated with tumor vascular endothelial growth factor and tumor aggressiveness. However, being influenced by many variables and since many different models are present in the literature, the quantitative approach still suffers from high output variability, poor clinical consistency and reproducibility. Qualitative DCE-MRI (qMRI) analyzes the time–intensity curve (TIC), involving the visual inspection and classification of TIC. The main weakness of qMRI is the ROI positioning that makes this approach operator dependent. Semi-quantitativeDCE-MRI is based on the analysis of TIC shape descriptors providing immediate data, related to the pathophysiology of the tumour. This approach could be more robust in clinical practice compared to quantitative o qualitative method, since many critical issues are reduced. However, semi-quantitative parameters do not show a direct analysis of physiological appearance [96, 97].

Quantitative DCE-MR parameters are derived from a pharmacokinetic model analysis and, on the basis of the mathematical model used, they reflect the underlying perfusion and/or permeability of the target tissue. *K*trans can have different physiologic interpretations depending on the balance between the blood flow and capillary permeability in the target tissue. If the contrast material uptake is flow limited, then *K*trans is related to the tissue perfusion [97]. Theoretically, hypervascular tumors are usually composed of a larger vascular space (vp) relative to the interstitial space (ve) and show a pattern of rapid arterial enhancement followed by washout, whereas a hypovascular tumor usually consists of a larger ve relative to the vp and shows progressive enhancement [97]. The liver is a dual-blood-supply organ that accepts blood from both the hepatic artery and portal vein. This unique physiological structure means that calculating the quantitative DCE-MRI parameters differs from that for organs with a single blood supply. Based on the characteristics of the dual blood supply, most quantitative analysis of DCE-MRI of the liver is currently carried out using the dual-inputtwo-compartment extended Tofts (DITET) model [98–100]. However, given that HCCs are mainly supplied by the hepatic artery, the relative benefits of the single-inputtwo-compartment extended Tofts (SITET) and DITET models remain unclear [100]. However, although the arterial blood supply is relatively increased in HCC compared with the portal blood supply, the tumor still has a dual blood supply. Several researches showed that compared with the SITET model, both the permeability and perfusion parameters of DITET correlated better with CD31- MVD and CD34-MVD, suggesting that the mathematical DITET model was more consistent with the microcirculation environment of HCC [100]. The permeability and perfusion parameters of the DITET model may thus be used to predict and evaluate the efficacy of targeted therapy and the response to transarterial chemoembolization in HCC, and for the diagnosis and classification of hepatic fibrosis and evaluation of liver function [100].

Ktrans is an important pharmacokinetic parameter to assess vascular permeability and therapeutic effects after non-surgical treatment in HCC. Several studies have suggested that a larger drop of Ktrans is correlated with favorable clinical outcomes after sunitinib or Floxuridine therapy [101, 102]. However, the most important role of quantitative DCE-MRI is the assessment of treatment efficacy in advanced HCC after ablative methods. These treatments may not produce a change in Ktrans because Fp and PS may change in opposite directions. On the other hand, Fp and PS may be changed in varying proportions. In these types of situations, it is important to understand which part of the vasculature is affected by the treatment [103].

### Diffusion weighted imaging-MRI

DWI offers functional quantitative data on the tissue’s microstructure by means of the water proton mobility differences and cellular density evaluation. Water diffusion mobility is linked to cell density, vascularity and viscosity of the extracellular apparent diffusion coefficient (ADC), and it’s possible to identify imaging biomarkers for fibrosis, tumor fluid and cell membrane integrity using a mono-exponential model or with diffusion and perfusion parameters in a bi-exponential model [104–114]. Using an Intravoxel Incoherent Motion method (IVIM) bi-exponential model to analyze DWI data, it is possible to obtain the pure tissue coefficient (Dt) linked only to diffusion water mobility, the pseudo-diffusion coefficient (Dp) linked to blood mobility, and the perfusion fraction (fp) [115–122]. The traditional DWI data analysis approach is founded on the hypothesis that voxel water diffusion has a single component and follows a normal Gaussian distribution, and that water molecules diffuse without any constraint. However, water molecule diffusion within biologic tissue exhibits non-Gaussian behavior [123–132]. Jensen et al. in 2005 reported a non-Gaussian diffusion model called Diffusion Kurtosis imaging (DKI) [123] used to analyze DWI data. This model includes the mean value of the kurtosis median coefficient (MK), which measures the tissue diffusion deviation from a Gaussian model, and the mean value of the diffusion coefficient (MD) with the correction of the non-Gaussian bias [123].

The role of DWI and functional parameters obtained by DWI in HCC has been assessed by different studies. Lee et al. [133] showed that DWI allowed to differentiate between HCCs and dysplastic nodules. They found that 86 HCCs (84.3%) showed hyperintensity on DWI, conversely, only 3 dysplastic nodules had this feature. Piana et al. [134] showed that restricted signal on DWI sequences and hyperenhancement during arterial phase were more sensitive than conventional vascular criteria. DWI could be used as a helpful tool for HCC in patients with chronic liver disease, since it can accurately detect HCC in patients with chronic liver disease regardless of the lesion size [134–136]. However, several researchers [137] have shown that DWI not allow to differentiate HCC from other hepatic lesions, since these solid lesions also have increased cellularity, showing ADC values that overlap with ADC values of HCC.

Recently, non-Gaussian diffusion behavior has been described in HCC and several authors have also evaluated the utility of the non mono-exponential models for the characterization and the assessment of treatment response in HCC patients [138–140]. However, the knowledge is still limited on which non mono-exponential model could more accurately evaluate the non-Gaussian DWI signal.

The pathological grade of HCC is connected to the prognosis, and it is one of the independent predictive features for recurrence and long-term survival after hepatic resection [141, 142]. However, it is challenging to define accurate preoperative grade using imaging modalities. Several studies [13, 117, 143–145] have assessed the role of several parameters by DWI and histological grade of HCC. Chen et al. [145], in a meta-analysis, found that for distinguishing well differentiated nodules from higher grades, DWI showed a low sensitivity (54%), high specificity (90%), and an excellent diagnostic performance. Conversely, in differentiating poorly differentiated nodules from lower grades, the sensitivity was 84%, the specificity 48%, showing a moderately high diagnostic performance. Granata et al. [117] found that DWI could be used to predict the histological grade of HCC; in fact, they showed that there was a good correlation between ADC and grading, between perfusion fraction (*fp*) and grading, and between tissue pure diffusivity (*Dt*) and grading.

### Bold-MRI

Hypoxia is emerging as a key factor of the aggressive tumor biology and the relative resistance to conventional as well as targeted therapies [146]. Several factors contribute to the low oxygen tension. Among these are its characteristically low microvascular density, rapid tumor growth which may result in impaired architecture of the tumor vessels, as well as the extensive desmoplastic stroma that exerts mechanical stress on the tumor vasculature and thus impairing tissue perfusion [146].

The only noninvasive imaging method that can reflect in vivo blood oxygen level is blood oxygen level- dependent (BOLD) functional magnetic resonance imaging (fMRI). BOLD MRI makes use of paramagnetic properties of deoxyhemoglobin and is mainly used for regional quantification of oxygenation [146]. The specific imaging mechanism is as follows: iron ions of deoxyhemoglobin contain unpaired electrons and have a paramagnetic property that shortens the transverse relaxation time of nearby protons. This is reflected in tissue T2-star (T2*) value, which is negatively correlated with the deoxyhemoglobin concentration [146]. With the evolution of hepatic fibrosis, progressive disruption of normal liver architecture is seen, which gives rise to regional and global changes in perfusion. Venous flow typically decreases due to increased resistance in the portal venous system, and may bypass the parenchyma completely via portosystemic venous shunts. Hepatic arterial flow commonly increases to counteract this effect. Impaired hemodynamic response and increased arterial blood flow to the liver may account for the elevated T2* response seen in diseased liver tissue. This is consistent with a breakdown in autoregulatory function, and is similar to the reduced hemodynamic response to carbogen challenge [147, 148]. Measuring native T2* or change in T2*, in response to a hyperoxic stimulus has been proposed as an intrinsic marker of tumor oxygenation, and T2* is proportional to the concentration of deoxyhemoglobin, which in turn relates to arterial blood pO2.

BOLD assessment is an useful tool in the hepatocarcinogenesis process so as after local treatment.

Petterson et al. [147] observed a larger but more variable response to oxygen challenge in HCC, which may be expected given variations in oxygenation and perfusion, even within the same tumor type. The initial pathogenesis of HCC involves an increased arterial supply; however, in late-stage HCC the arterial blood supply may decrease. This variability in blood supply, and the consequent proportions of oxyhemoglobin, could be a source of variability in the observed BOLD response [147].

Tumoral changes caused by TACE such as tumor vascularity, blood supply ratio of the hepatic artery to the portal vein, and aerobic metabolic activity may be assessed by BOLD-fMRI [149]. This technique may thus serve as a novel and useful noninvasive functional biomarker for assessing the early T2* changes post-TACE. TACE reduced the BOLD response in the cancerous area by blocking the blood supply to the tumor [149]. In preliminary rabbit VX2 liver tumor oxygenation experiments, Rhee et al. demonstrated a statistically significant decrease in the T2* value after TACE with polyvinyl alcohol particles [150]. Choi et al. reported that TACE induced a significant reduction in the ∆ R2* values of tumors on the first day after the procedure [151].

### Radiomics analysis

Radiomics consists of the extraction of several parameters by radiological data that can provide information about tumor phenotype as well as the cancer microenvironment [152–160]. Radiomics, when combined with other data linked to patient outcome, can produce precise evidence-basedclinical-decision support systems. The main task is to combine and to collect differents multimodal quantitative data with a mathematical method in order to provide clear and robust clinical parameters and to allow outcome prediction [68]. The idea of radiomics is that the quantitative variables are more sensitively correlated with various clinical endpoints compared with qualitative radiologic and clinical data. Radiomics offer outstanding benefits over qualitative imaging assessment, since this is clearly limited by the resolution of radiologist ‘eyes [161–164]. A radiomic information extension can be obtained by adding genomics data (radiogenomics); in fact, genomic markers such as microRNA expression, have been shown associated with treatment response, metastatic spread and prognosis that could offer personalized and precision medicine [68]. Radiogenomics could perform patient selection for different cancer therapy, predict therapy, address potential therapy resistance (chemotherapy and/or radiation-therapy) and select patients with poor prognosis [68].

The framework of radiomic analysis mainly involves four phases: acquisition of the image, identification of a region of interest (manually or automatically), segmentation, and feature selection, model building, and classification. The approaches applied to evaluate radiomic traits are classified into three types including statistics-based, model-based, and transform-based [68].

To date, several studies have evaluated the radiomic or radiogenomic traits and factors affecting the survival of patients with HCC after chemotherapy, resection, interventional treatments, and transplantation through non- invasive imaging, and have highlighted the significance of non-invasive method in evaluating these prognostic factors [165]. The radiomic studies, assessing the relationship between imaging traits and clinical characteristics including survival, recurrence, or treatment response of HCC, have been greatly studied by CT. By contrast, relatively few studies have dealt with these relationships by MRI images. This may be due to the difficulty of providing standardization of MR images compared with CT imaging. In fact, MRI provides the advantage of high-contrast structural and functional information illustrating soft-tissue characteristics, and the technique is superior in assessing the tumor’s metabolism and proliferation with higher accuracy. Metabolic imaging using 18-fluoro-deoxyglucose positron emission tomography (FDG-PET) has also been used to demonstrate the prognostic value of radiomics in HCC [166].

The areas of greatest interest were related to the characterization of the lesions [167–173] and the evaluation of the response to therapy [174–182].

For the differentiation between HCC and benign hepatic lesions, a CT-based radiomics nomogram, which incorporated the rad-score and clinical factors showed an area under the receiver operating characteristic curve (AUC) of 0.917 for differentiating FNH from HCC [183]. An MRI-based study in 369 patients with 446 lesions (HCC 222, hemangioma 224) reported an AUC of 0.89 (sensitivity 0.822, specificity 0.714) for differentiating between HCC and hemangioma using images with in-phase, out-phase, T2-weighted, and diffusion-weighted imaging sequences [184]. In addition, according to a more recent study with both CT and MRI, fusion models that simultaneously integrated clinical characteristics achieved average AUCs of 0.966 (CT) and 0.971 (MRI), with 10-foldcross-validation to differentiate hepatic epithelioid angiomyolipoma from HCC and FNH [185]. Furthermore, a multicenter retrospective cohort study performed in 178 cirrhosis patients (with indeterminate liver nodules including other malignant lesions as cholangiocarcinoma and metastasis, regenerative nodule, hemangioma and FNH) reported an AUC of 0.66 to diagnose HCC using triphasic contrast-enhanced CT, and suggested the benefit of AI to enhance clinicians’ decisions by identifying a subgroup of patients with high HCC risk [169].

Several studies suggest the effect of radiomics-based data to help make treatment decisions by the noninvasive prediction of the response to TACE and immune-oncologic features [174–182]. Thus, if the therapeutic direction is considered to be unresponsive to TACE, it may be changed to the use of molecular targeted agents (i.e., sorafenib). However, the practical benefit of radiomics-based findings needs to be validated by additional studies in a prospective setting.

Although radiomics is not a panacea for clinical management, it is an up-and-coming and highly relevant method in cancers and in diseases other than malignancy such as neurological and vascular pathologies [156, 186, 187].

### Diagnostic management-Li-RADS

HCC may be noninvasively diagnosed by imaging findings alone, often without biopsy [188]. To standardized imaging and reporting, the American College of Radiology (ACR) developed LI-RADS at CT or MRI in patients at risks for HCC in 2011, which has been refined and expanded over multiple updates to version 2018 till now to consist with clinical practice, such as introducing the concept of LR-OM for non-HCC malignancies in 2013, which was renamed as LR-M in 2014 [189–191]. Besides, CEUS highlights itself with a real-time observation, for which ACR established the CEUS LI-RADS in 2016 and further revised LR-M observations in 2017 [192–194]. Several studies showed the value of LR-M observation for differentiating non-HCC malignancies from HCC. An et al. [195] found that CT and MRI showed comparable capabilities for distinguishing non-HCC malignancies from HCC based on CT/MRI LI-RADS, with pooled accuracies of 79.9 and 82.4% for categorizing LR-M. Kim et al. [196] demonstrated that non-HCC malignancy could be distinguished from HCC at a sensitivity of 89% and a specificity of 48% in patients with liver cirrhosis by using the LR-M criteria of CT/ MRI LI-RADS v2018 at gadoxetate-enhanced MRI. Zheng et al. [197] validated the CEUS LI-RADS by showing a sensitivity of 89% and a specificity of 88% for the LR-M category to distinguish non-HCC malignancy from HCC. Due to good diagnostic performance, CT/MRI LI-RADS was integrated into HCC guidance from American Association for the Study of Liver Diseases (AASLD) in 2018 [198]. However, AASLD does not accept CEUS as a diagnostic technique but a second-line technique after CT or MRI [198].

LI-RADS offers four individual imaging algorithms designed for different clinical contexts: *(a)* US LI-RADS for surveil- lance, *(b)* CT/MRI LI-RADS for diagnosis and staging, *(c)* CEUS LI-RADS for diagnosis, and *(d)* treatment response LI-RADS to assess response to local-regional therapies [198].

LI-RADS defines eight unique diagnostic categories based on imaging appearance that reflect the probability of HCC or malignancy with or without tumor in vein. The category LR-NC (not categorizable) is applied when image omission or degradation precludes categorization. The categories LR-1 (definitely benign) and LR-2 (probably benign) range from simple cysts to LR-2 distinctive nodules. An LR-2 distinctive nodule is defined by its size (< 20 mm) and the absence of any major features of HCC, any features of LR-M, or any ancillary features of malignancy [198]. LR-3 (intermediate probability of HCC) includes some perfusion alterations that have a nodular shape and true nodules with one or two malignant features. The malignant categories range from probable to definite malignancy and include LR-4 (probably HCC), LR-5 (definitely HCC), LR-M (probably or definitely malignant, not specific for HCC), and LR- TIV (malignancy with tumor in vein) [198].

The LI-RADS lexicon divides imaging features into major features, LR-M features, and ancillary features. Major features include nonrim APHE, nonperipheral “washout” appearance, enhancing “capsule” appearance, size, and threshold growth [198]. LR-M features include a targetoid or nontargetoid mass with one or more of the following findings: infiltrative appearance, marked diffusion restriction, necrosis, or other features suggestive of non-HCC malignancy [198]. Ancillary features are divided into those favoring benignity, those favoring malignancy, and those favoring HCC. Their use is optional, and they may be used to up or downgrade a category by one, but they cannot be used to upgrade to LR-5 [198].

Multiphase contrast-enhanced imaging is necessary to assess LI-RADS features. Intravenous extracellular contrast agents are used for CT, while those used for MRI may be extracellular or hepatobiliary. Both gadoxetate disodium and gadobenate dimeglumine may be used in hepatobiliary phase imaging. For treatment-naive patients undergoing CT, unenhanced imaging is optional; however, it is required in the post treatment setting for CT and all MRI studies. Late arterial phase is strongly preferred over early arterial phase [198].

The choice of modality (CT or MRI) and MRI contrast agent (extracelllar or hepatobiliary) depends on patient, institutional, and regional factors. LI-RADS does not endorse any particular imaging method, rather, it provides guidance on technique, terminology, interpretation, and reporting [198].

CEUS is most suitable for problem solving, categorizing individual observations, and differentiating tumor in vein from bland thrombus rather than for staging the entire liver. CEUS requires expertise and specialized equipment [198].

### Ablation techniques assessment

The primary endpoint of ablation therapy is to obtain a complete necrosis (similar to R0 resection) of liver tumors that is linked to create a safety margin of at least 10 mm round the external margin of the lesion. However, the effectiveness of the treatment is linked to numerous features, such as tumor size, location, blood flow, and equipment utilized [14]. RFA and MWA are hyperthermic techniques [14, 199]. RFA produces necrosis thanks to thermocoagulation. With RFA, the zone of active tissue heating is restricted to a few millimetres nearby to the electrode, with the residue of the target being heated via thermal conduction [14, 199]. Consequently, the treatment efficacy is closely related to the lesion size, and the maximum result is obtained for target less than 3.5 cm [14, 199]. Additionally, some tissue features, such as electrical conductivity, thermal conductivity, dielectric permittivity, and blood perfusion rate, have effect on the efficacy of RFA procedure.

MWA uses the dielectric effect, which occurs when an imperfect dielectric material is subjected to an alternating electromagnetic (EM) field, generating a larger area of active heating (up to 2 cm close the antenna) allowing more homogeneous necrosis in the target zone, compared to RFA [199]. Conversely, to RFA and MWA, IRE and ECT are non-thermal techniques that cause ablation changing cell membrane permeability thanks to an induced electric field (electroporation).

IRE is considered as a direct ablation tool, since electroporation is used in irreversible manner [199]. Short high-voltage electric current fields cause the irreversible permeabilization of the lipid bilayer, the disruption of the cellular homeostasis, and the stimulation of apoptotic pathways, causing death of neoplastic cells [199]. Taking into account its mechanism of action, IRE can protect surrounding structures, such the vessels and bilir tree [199].

Among all the ablative procedures, RFA is a frontline technique for HCCs smaller than 20 mm [14]. Several studies have evaluated the efficacy of RFA with respect to resection and have established that RFA is a noninvasive and effective ablative treatment [200–204]. Although these studies focused on RFA as a stand-alone therapy contained valuable information regarding treatment safety and response, they lacked sufficient follow-up to define important long-term outcomes such as survival. Only recently have survival data become available on RFA-treated patients with HCC. Large clinical series from Europe, the U.S., and Asia have demonstrated 5-yearpost-RFA survival rates between 33 and 55%, comparable to those seen in series of hepatic resection [205].

With the microwave technology progress and a continuously cooled electrode development, MWA has recently been used more recurrently in treatment of HCC [206–208]. According to the pubblished data, overall survival, local recurrence, complication rates, disease-free survival, and mortality in patients with HCC treated with MWA (compared with RFA) vary between 22 months for focal lesion > 3 cm (vs. 21 months) and 50 months for focal lesion ≤3 cm (vs. 27 months), between 5% (vs. 46.6%) and 17.8% (vs. 18.2%), between 2.2% (vs. 0%) and 61.5% (vs. 45.4%), between 14 months (vs. 10.5 months) and 22 months (vs. no data reported), and between 0% (vs. 0%) and 15% (vs. 36%), respectively [14].

Postablation imaging is necessary to assess the treatment results, to monitor evolution of the ablated tissue over time, and to evaluate for complications [209]. Post-thermal treatments, imaging should be performed at regularly scheduled intervals to assess treatment response and to evaluate for new lesions and potential complications. Although there is no widely accepted post-treatments imaging surveillance protocol, the protocol should include 1-, 3-, 6-, 9-, and 12-monthcontrast-enhanced CT or MRI follow up. Periablation enhancement that occurs as a result of inflammation in the surrounding parenchyma and that could depict residual disease gradually decreases over time [210].

On unenhanced CT performed closly after thermal treatment, the ablation zone is larger than the initial target and is hypoattenuating or heterogeneously hyperattenuating because of coagulative necrosis and hemorrhagic products; however, occasionally no visible changes may be discernible on CT [210]. At the first follow-up CECT or MRI evaluation, the ablation zone can be spherical, oval, or oblong dependent on the number and type of electrodes used. For lesion located between blood vessels, the shape of the ablation zone post RFA can be irregular because of the “heat sink” effect. On unenhanced CT, the ablation zone becomes more homogeneously hypoattenuating over time. On MRI, the treated leion is heterogeneously or peripherally hyperintense on T1-weighted images and heterogeneous or hypointense on T2-weighted because of coagulative necrosis, hemorrhagic products, and dehydration. These changes can persist for variable periods of time; the imaging appearances change as the blood products evolve and also become more homogeneous over time. Marked hyperintensity on T2-weighted imaging suggests liquefactive necrosis or biloma formation. On CECT and MRI, the ablation zone is well demarcated and no enhancement suggests a lack of viable tumor [210]. CEUS has been employed in the early assessment of the ablated HCC, being comparable to CT and MRI in the detection of residual viable tumor [211]. However, the potential role of CEUS in the follow-up of the patient with a successful ablation has not been adequately investigated. CEUS is indicated in the assessment of local tumor progression when follow-up CT or MR are contraindicated or not conclusive and that, in addition to CT and/or MR, CEUS may be used in follow-up protocols. CEUS has the limitation of being unable to explore the entire liver during the arterial phase, which is a rather limited time frame. Consequently, while CT and MRI allows to scan the whole liver during the arterial phase and to rescan it during the portal and late phase achieving a multiphase study, CEUS may miss a transiently hyperperfused lesion just because not exploring that given liver area in the appropriate moment [211].

IRE is a relatively new minimally invasive image-guided technique for the interventional oncologic treatment of soft tissue tumors. IRE offers several potential advantages over thermal ablation approaches, specifically, a relatively short ablation procedure and the ability to ablate tumors adjacent to large blood vessels. Thus, IRE may be well suited for the ablation of solid organ tumors such as primary or metastatic lesions in the liver, especially those located near blood vessels, bile ducts, and nerves [212]. However, one possible disadvantage of IRE may be its lower success rate for complete local tumor eradication. This may be attributable to the fact that the optimal timing of image-basedfollow-up and the most suitable imaging modality for follow-up have yet to be determined because IRE therapy has only recently been introduced. With regard to this point, another problem is that the contrast-enhanced imaging characteristics of liver tumors that have been successfully treated by IRE ablation differ from those observed following RFA. Specifically, unlike RFA, persistent enhancement of the peritumoral liver parenchyma is observed within the IRE ablation zone, and thus the ablated margin is not clear in IRE. These are crucial factors for identifying residual tumor and for planning re-treatment [212]. Sugimoto et al. evaluated the diagnostic performance of CEUS, CECT and EOB-MRI in the assessment of immediate response to IRE in liver lesions. They found, during the arterial phase of CEUS, EOB-MRI, and CECT images five categories of post IRE lesions, according to their enhancement patterns. The areas under the ROC curve for CEUS were significantly higher than those for EOB-MRI and CECT. The sensitivities and specificities were higher with CEUS than with EOB-MRI and CECT. However, the differences were not statistically significant. Ths study showed that CEUS was the ideal modality for evaluating the treatment response to IRE in the subacute phase (i.e., within 1 week after IRE). Continuous real-time observation of hemodynamic changes in ablated lesions is possible with CEUS because microbubbles are pure intravascular tracers that remain in the blood pool. CEUS is, therefore, very sensitive to the presence of a residual functional vascular bed in the ablated area and can provide information concerning the vessels remaining in the treated area. Moreover, CEUS can be performed immediately after the IRE procedure without difficulty [212].

**Fig. 9:**
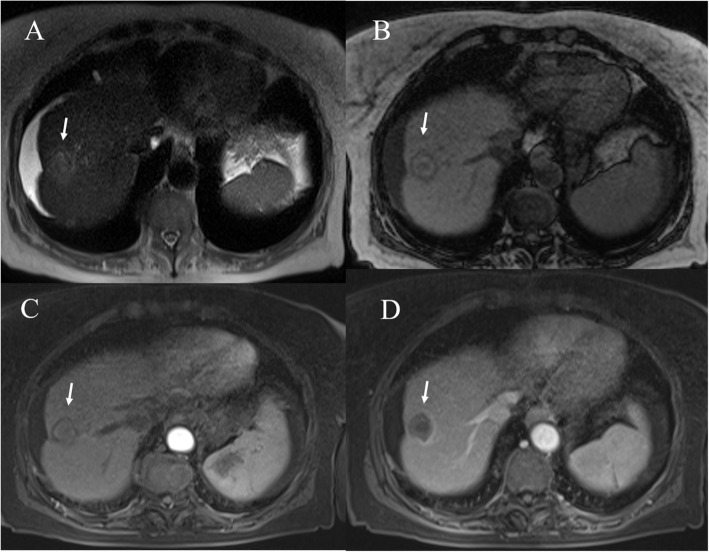
Post RFA MRI assessment of HCC on VIII hepatic segment. The ablated lesion is non viable with isointense signal in T2-W (**A**) sequence, targetoid appearance in T1-W (**B** and **C**) sequences and no hypeeenhancement during artierial phase (**D**) of contrast study

**Fig. 10:**
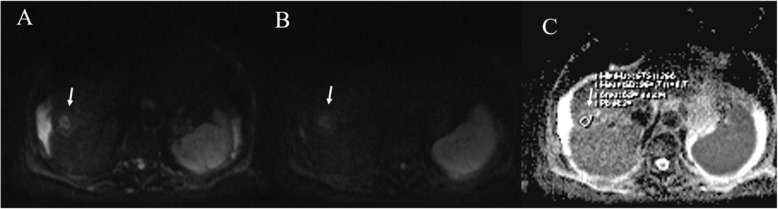
The same patient of Fig. 9. DWI assessment. The ablated area shows targetoid appearance in b50 s/mm2, in b 800 s/mm^2^ and ADC map

The contrast enhancement imaging characteristics of successfully IRE-ablated liver tumors are different from those seen in RFA. A previous study in a patient with HCC treated by IRE reported that despite persistent enhancement of peritumoral liver parenchyma within the IRE ablation zone, the tumor itself was clearly demarcated by a devascularized area in comparison to surrounding unablated or ablated liver parenchyma [213]. In our previous study we dscribed the MRI findings of HCC treated with IRE at 1-monthfollow-up [214]. Ablation zones showed a round shape in 20 of 24 treated lesions. Those zones located underneath the hepatic capsule had an oval configuration (4/24; 17%). The ablation zones increased in size by 10% compared with their initial size on pretreatment imaging. On T1-weighted images, all lesions (100%) showed a nonhomogeneous signal, with a hyperintense central core and a hypointense peripheral rim. On T2-weighted sequences, the signal from the necrotic ablation zone was heterogeneously hypointense (Fig. 9). The residual tumor tissue appeared as a peripheral portion that was hypointense on the T1-weighted images and hyperintense on the T2-weighted images. On DWI, twenty out of 24 treated lesions (83%) showed restricted diffusion. On the other hand, when the lesions were not clearly visible at a b value of 0 s/mm^2^ (4/24; 17%), there was no signal detectable at a b value of 800 mm/s^2^, where only the rim showed restricted diffusion (targetoid appearance) (Fig. 10). The ADC values did not show any statistically significant difference for each single lesion evaluated between baseline and at 1 month, with a large overlap between the ADC values recorded before and after IRE. During the dynamic sequences and the liver-specific phases, the treated area showed hypointensity signal. The ablation zones containing residual viable tumor (2/24; 8.3%) showed contrast enhancement during the arterial phase and portal phase washout. The residual tumor tissue appeared as hypointense, although to a lesser degree than the necrotic portion, in the hepatobiliary phase. In four peripheral treated lesions (17%), there was capsular retraction. Six out of 20 patients (30%) showed a THID area within the normal liver parenchyma adjacent to the treated lesions. Two out of the 20 patients (10%) had no concentration of liver-specific contrast medium around the ablation zone [214].

Few studies describe the radiological findings of ECT on treated liver lesions. The response of a tumour after ECT is slow due to its mode of action, i.e., due to slow killing of the dividing cells that occurs due to the internalization of the bleomycin by electroporation. The treated tumor gradually changes into fibrotic tissue during a period of 4 months [215]. Boc et al., assessed US changes in the liver treated ECT tumors [216]. During the 1st phase took up to 5 min after the delivery of electric pulses. The hyperechoic microbubbles were observed along the electrode tracks and were visible immediately i.e. within a few seconds after the pulses were triggered and later within the entire ablation zone. During the 2nd phase, after 5–15 min, microbubbles were distributed throughout the treated tumor, and the tumor became hyperechoic and surrounded by a hypoechoic zone. The hypoechoic zone (5–15 mm wide) represents the electroporated area within the normal liver tissue represents the treatment safety margin. Four days after ECT, US of the metastasis in patient #1 she presented with an 18-mm hyperechoic formation surrounded by a 5-mm hypoechoic area, which is most likely the oedematous area of the liver parenchyma, or the safety margin. Five months after ECT, US and MRI showed the metastasis in *patient 1* as a fibrotic residuum without the hypoechoic rim. The size of the metastasis was not significantly reduced. The appearance was as a complete response, which was also present 7 months after ECT; however, at that time, the size was significantly reduced to 11 mm from the original 20 mm in the greatest diameter, indicating the slow resorption of the treated metastasis. Similar MRI findings were shown in *patient 2*, 3 months after ECT in which the treated metastasis was reduced from 19 to 13 mm in diameter [216].

Tarantino et al. treated with ECT a prospective case series of patients with liver cirrhosis and Vp3-Vp4- portal vein tumor thrombus (PVTT) from HCC, in order to evaluate the feasibility, safety and efficacy of this treatment. Post treatment intraoperative CEUS demonstrated complete absence of enhancement of the thrombosis and of the treated HCC nodule in all cases. The follow-up ranged from 9 to 20 mo (median, 14 mo). In these two patients, CEUS and CT confirmed complete patency of the vessel without any intravascular or perivascular recurrence during follow-up. In three patients, CT and CEUS showed permanent complete thrombosis with a persistent, shrinked, avascular thrombus into the treated vessels. In all three cases, no intravascular or perivascular enhancement consistent with residual tumor or local recurrence was detected at CT and CEUS during follow- up. In the remaining patient, 24 h post-treatment CEUS showed absence of enhancement of the treated thrombus. However, the patient was lost to follow-up because of death from gastrointestinal hemorrage 5 weeks after ECT treatment [217].

It is clear that, considering therapeutic responses to treatments, imaging data are sometimes complicated to understand because it depend on anatomic location, on the method of act of given therapy, on the morphological and functional criteria that are used for each imaging modality. In this setting, imaging observations depend highly on the type and the method of therapy delivery, the timing of treatment, and the imaging technique being used to observe the effects. Therefore, an evaluation based only on dimensional data is not appropriate to assess the efficacy of such complex treatments, since not always a positive response to treatment is linked to a size reduction; furthermore dimensional criteria do not allow the differentiation of the fibrotic tissue from the residual tumor [218]. The LI-RADS treatment response algorithm applies to multiphase CT or MRI used to assess response after local-regional therapy, which includes percutaneous therapy (eg, ethanol and radiofrequency or microwave ablation), transcatheter therapy (eg, transarterial chemoembolization or radioemboliza- tion), and external beam radiation therapy. The algorithm also applies to observations at the surgical margin after resection of lesion. The LI-RADS treatment response algorithm does not apply to systemic chemotherapies, targeted, and/or immunologic therapies [198].

LI- RADS treatment response category codes reflect the relative probability of tumor viability after local-regional therapy to guide management decisions. Similar to the modified Response Evaluation Criteria in Solid Tumors (mRECIST) [219], the LI-RADS algorithm is based on unidimensional measurements of the largest enhancing component of a treated tumor, excluding areas of nonenhancement.

## Conclusion

HCC may be noninvasively diagnosed by morphological and functional imaging. To day, the vascular assessment of focal hepatic nodule allow to identy HCC nodules, so that multiphase contrast-enhanced imaging is necessary during diagnostic phase. Intravenous extracellular contrast agents are used for CT, while those used for MRI may be extracellular or hepatobiliary. Both gadoxetate disodium and gadobenate dimeglumine may be used in hepatobiliary phase imaging. For treatment-naive patients undergoing CT, unenhanced imaging is optional; however, it is required in the post treatment setting for all CT and MRI studies. Regarding to contrast study protocol, late arterial phase is strongly preferred over early arterial phase.

Although the choice of modality (CT, US/CEUS or MRI) and MRI contrast agent (extracelllar or hepatobiliary) depends on patient, institutional, and regional factors. However, MRI allows to link morfological and functional data in the HCC evaluation.

Postablation imaging is necessary to assess the treatment results, to monitor evolution of the ablated tissue over time, and to evaluate for complications. Post-thermal treatments, imaging should be performed at regularly scheduled intervals to assess treatment response and to evaluate for new lesions and potential complications. At the first follow-up CECT or MRI evaluation, the ablation zone can be spherical, oval, or oblong dependent on the number and type of electrodes used.

## Data Availability

All data are reported in the manuscript.
